# Simplifying the Experimental Detection of the Vortex Topological Charge Based on the Simultaneous Astigmatic Transformation of Several Types and Levels in the Same Focal Plane

**DOI:** 10.3390/s22197365

**Published:** 2022-09-28

**Authors:** Pavel A. Khorin, Svetlana N. Khonina, Alexey P. Porfirev, Nikolay L. Kazanskiy

**Affiliations:** 1 Department of Technical Cybernetics, Samara National Research University, 443086 Samara, Russia; 2 Image Processing Systems Institute of RAS—Branch of the FSRC “Crystallography and Photonics” RAS, 443001 Samara, Russia

**Keywords:** multi-channel diffractive optical element, astigmatic transformation, vortex beam, topological charge

## Abstract

It is known that the astigmatic transformation can be used to analyze the topological charge of a vortex beam, which can be implemented by using various optical methods. In this case, in order to form an astigmatic beam pattern suitable for the clear detection of a topological charge, an optical adjustment is often required (changing the lens tilt and/or the detection distance). In this article, we propose to use multi-channel diffractive optical elements (DOEs) for the simultaneous implementation of the astigmatic transformations of various types and levels. Such multi-channel DOEs make it possible to insert several types of astigmatic aberrations of different levels into the analyzed vortex beam simultaneously, and to form a set of aberration-transformed beam patterns in different diffraction orders in one detection plane. The proposed approach greatly simplifies the analysis of the characteristics of a vortex beam based on measurements in the single plane without additional adjustments. In this article, a detailed study of the effect of various types of astigmatic aberrations based on a numerical simulation and experiments was carried out, which confirmed the effectiveness of the proposed approach.

## 1. Introduction

Vortex beams are special structured laser beams, the special properties of which have been actively studied for several decades [1,2,3,4,5,6]. The most important among the special properties is the presence of an orbital angular momentum [7,8,9,10,11], which is determined by the order of the optical vortex, also called the topological charge (TC). A distinctive feature of an optical vortex beam is the presence of vortex phase singular points, at which the phase is not defined and the amplitude is zero. Vortex beams have been effectively used in various applications, including optical information transmission [12,13,14,15,16,17], optical trapping and manipulation [18,19], probing [20,21] and many others. Therefore, the development of convenient and simple methods for determining the characteristics of vortex beams seems to be extremely important.

There are various methods for determining the TC of a vortex beam based on matched filtering [17,18,19,20,21,22,23,24,25,26], the use of special elements ensuring beam sorting [27,28,29,30] and also with an astigmatic transformation [31,32,33,34,35,36,37,38]. The simplest way for an astigmatic transformation is applying a cylindrical or tilted lens [39,40,41] as well as a tilted or astigmatic axicon [42,43,44]. Inclined/tilted optical elements also insert an astigmatic transformation into generated vortex beams [45,46]. Astigmatic transformations can be implemented using anisotropic crystals [47,48,49,50].

There is also a method based on curvilinear and parabolic-line diffraction grating [51,52,53,54], which actually corresponds to the insertion of a certain astigmatic transformation in the diffraction orders of a one-dimensional grating. We propose a more general approach providing several aberration transformations of various types and magnitudes (levels) in a 2D set of diffraction orders.

The magnitude (level) of an astigmatic transformation of abeam depends on various factors, including both the characteristics of optical elements and systems (focal length, tilt angle and degree of anisotropy) [39,45,47] and the properties of the beam, including the TC value [46,55]. Therefore, in order to form an astigmatic beam pattern suitable for the clear detection of the TC, an optical tuning (changing the lens tilt and/or the detection distance) is often required. In this paper, for the simultaneous implementation of astigmatic transformations of various types and levels, we propose to use multi-channel diffractive optical elements (DOEs) [56,57,58]. To design a multi-channel DOE matched with several aberrations, we use the method of spatial carrier frequencies [59]. Thus, a single multi-channel DOE can simultaneously generate several different beams at given points of the focal plane in accordance with the spatial carrier frequencies. We emphasize that such a DOE is not a simple two-dimensional grating. In particular, as shown numerically in [58], a 25-channel DOE allows for the insertion of several types of astigmatic aberrations of different levels into the analyzed vortex beam simultaneously, forming a set of aberration-transformed beam patterns in different diffraction orders in one detection plane. The proposed approach greatly simplifies the analysis of the characteristics of a vortex beam based on measurements in the single plane without additional adjustments.

The article presents a detailed study of the effect of various types of astigmatic aberrations based on a numerical simulation and experiments, which confirmed the effectiveness of the proposed approach.

## 2. Methods

### 2.1. Theoretical Foundations

The astigmatic transformation in the classical form can be written as follows [60]:

(1) $$\psi_{ast}\left(x,y;\beta \right)=\left(x^{2}-y^{2}\right)\cos2\beta -2xy\sin2\beta$$

where β is the rotation parameter.

On the basis of Expression (1), special cases can be considered, for example:

(2) $$\begin{array}{l} \psi_{ast}\left(x,y;0\right)=\left(x^{2}-y^{2}\right),\\ \psi_{ast}\left(x,y;\pi /4\right)=-2xy. \end{array}$$

Astigmatic transformations using a cylindrical or inclined lens are also known [39,40,41]. In this case, the transformation can be described as follows:

(3) $$\psi_{tilt}\left(x,y\right)=a_{x}x^{2}+a_{y}y^{2}$$

Since Transformations (1) and (3) do not reduce to each other, in this paper, we considered a more general concept of an astigmatic transformation in the following form:

(4) $$\psi_{gen}\left(x,y\right)={\left(a_{x}x+a_{y}y\right)}^{2}={\left(a_{x}x\right)}^{2}+{\left(a_{y}y\right)}^{2}+2a_{x}a_{y}xy$$

Assuming the parameters $a_{x},a_{y}$ to be complex, we could obtain both options of Equations (1) and (3).

It should be noted that the astigmatic transformation also takes place in the case of wavefront astigmatic aberrations. Such aberrations are usually described using the Zernike polynomial basis [61,62]:

(5) $$Z_{nm}^{}\left(r,\varphi \right)=\sqrt{\frac{n+1}{\pi }}R_{n}^{m}\left(r\right)\left\{\begin{array}{c} \cos\left(m\varphi \right)\\ \sin\left(m\varphi \right) \end{array}\right\}$$

where $R_{n}^{m}\left(r\right)$ are radial Zernike polynomials normalized in the circle of radius $r_{0}$:

(6) $$R_{n}^{m}\left(r\right)=\sum_{s=0}^{\left(n-m\right)/2}\frac{{\left(-1\right)}^{s}\left(n-s\right)!}{s!\left(\frac{n+m}{2}-s\right)!\left(\frac{n-m}{2}-s\right)!}{\left(\frac{r}{r_{0}}\right)}^{n-2s}$$

Note that the aberrations of the astigmatic type include the functions from Equation (5) with index *m* = 2. Table 1 lists several Zernike functions of Equation (5), corresponding to astigmatic aberrations.

Table 1 shows that the Zernike functions corresponding to astigmatic aberrations contained terms of the form $\left(x^{n}-y^{n}\right)$, which is more general than the expression of Equation (4). Table 1 also shows the phase distribution of the corresponding wavefront with values in the range from 0 to 2π.

Table 2 shows the correspondence with particular cases of astigmatic transformations of Equation (4) and Zernike functions. Table 2 presents that special cases of the astigmatic transformation in Equation (4) could be uniquely represented as a combination of astigmatic aberration *Z*_2,±2_ and defocus *Z*_2,0_ (corresponding to a shift from the focus plane). Thus, the use of cylindrical lenses and other types of astigmatic transformations could lead to the need to select the best detection distance. However, this action can be replaced by changing the aberration magnitude (level).

The study of this issue based on a numerical simulation is given in the next section.

### 2.2. Simulation of Astigmatic Transformations

For the analyzed vortex beam, the Laguerre–Gaussian modes were considered [63,64,65]:

(7) $$\Psi_{pl}(r,\varphi )=\sqrt{\frac{2p!}{\pi (p+\left|l\right|)!}}\exp\left(-\frac{r^{2}}{\sigma^{2}}\right)\cdot {\left(\frac{\sqrt{2}r}{\sigma }\right)}^{\left|l\right|}L_{p}^{\left|l\right|}\left(\frac{2r^{2}}{\sigma^{2}}\right)\exp(il\varphi )$$

where $L_{p}^{l}(x)$ is the generalized Laguerre polynomial [63], σ is the parameter of the Gaussian beam and index *l* corresponds to the TC of the vortex beam.

The Fresnel transform was used to simulate the effect of certain types of astigmatic aberrations on vortex beams:

(8) $$F(\rho ,\theta )=-\frac{ik}{2\pi z}\exp(ikz)\int_{0}^{\infty }\int_{0}^{2\pi }f(r,\varphi )\exp\left\{i\frac{k}{2z}\left[r^{2}+\rho^{2}-2r\rho \cos(\theta -\varphi )\right]\right\}rdrd\varphi$$

where k=2π/λ is the wave number, λ is the radiation wavelength, f(r,φ) is the input function and *z* is the distance from the input plane. The following distribution was used as an input function in this section:

(9) $$f(r,\varphi )=\Psi_{0,l}(r,\varphi )\exp\left[ik\alpha Z_{n,2}(r,\varphi )\right]\exp\left(-ik\frac{r^{2}}{2z_{0}}\right)$$

The function $\exp\left[ik\alpha Z_{n,2}(r,\varphi )\right]$ in Equation (9) corresponds to the insertion of astigmatic aberrations of various types (Table 1) with various magnitudes (levels) determined by the parameter α into a vortex beam $\Psi_{0,l}(r,\varphi )$ with TC equal to *l*. The optical beam focusing was implemented with the use of a lens $\exp\left(-ik\frac{r^{2}}{2z_{0}}\right)$ with focal length *z*_0_.

Table 3 shows the results of modeling the astigmatic transformation of vortex beams $\Psi_{0,l}(r,\varphi )$ for different values of TC when various astigmatic aberrations $Z_{n,2}(r,\varphi )$ were used. The intensity distributions in the focal plane were shown. The following parameters were used in the calculation: λ = 532 nm; σ = 0.15 mm; focal length *z*_0_ = 300 mm.

According to the results shown in Table 3, the formation of astigmatic patterns was clearly visible, which allowed one to fairly confidently determine the TC value (vortex order *l*) with the number of dark lines in the intensity distribution. Note that this approach works correctly both with optical vortices with an even and an odd TC. There were no fundamental differences in the astigmatic pictures. There was an even or odd number of minimums on the intensity distribution.

Table 4 shows similar simulation results, taking into account the displacement of the detection plane from the focal plane by a distance Δ*z*.

Interestingly, not only the sign of the topological charge, but also the type of aberration affected the slope of the formed pattern. It should be noted that high-order astigmatic aberrations (*n* ≥ 6) led to the formation of less clear and inconvenient visual analysis patterns (Table 3). However, the third-order astigmatism (*n* = 6) demonstrated a resistance to defocusing and good results for low values of TC (Table 4).

Table 4 shows that the resistance of the formed astigmatic patterns to defocusing depended both on the value of the TC and on the type of astigmatic transformation. As a rule, as the TC value *l* increases, the resistance to defocusing decreases. This drawback can be compensated by increasing the level of astigmatic aberration due to parameter α.

## 3. Proposed Approach Based on Multi-Channel DOEs

### 3.1. Principle of Operation

In order to not have to perform additional optical adjustments of the optical system, it was proposed to carry out simultaneous astigmatic transformations of various types and levels based on the use of multi-channel DOEs [56,57,58].

**Figure 1:**
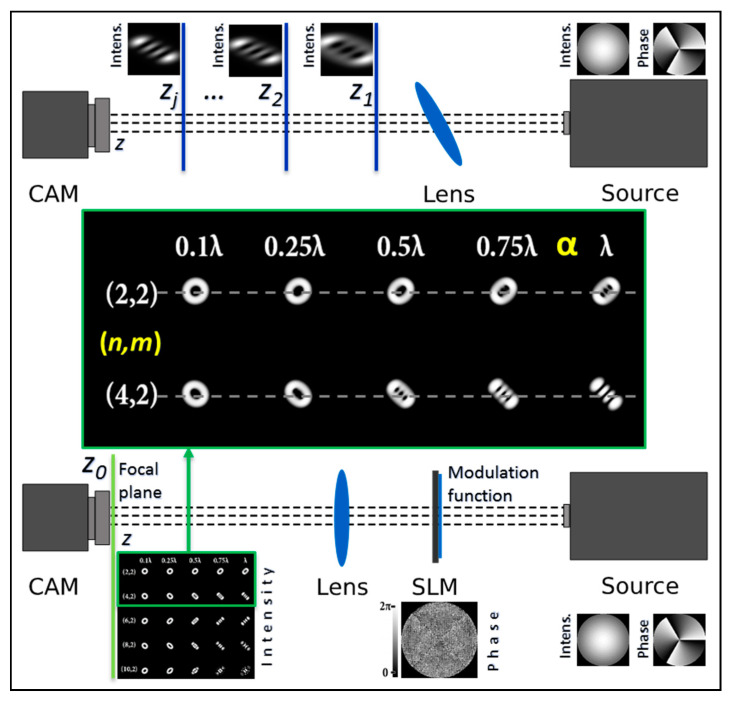
Principle of operation for determining the vortex TC (*l* = 3) in a standard way (upper part) using a tilted lens and detecting the intensity distribution in several planes (*z*_1_, *z*_2_, …, *z_j_*) and the proposed approach (lower part) based on multi-channel DOE matched with astigmatic aberrations $\exp\left[ik\alpha Z_{n,2}(r,\varphi )\right]$ of different levels α in a single focal plane *z*_0_.

The multi-channel DOE allowed for the introduction of several types of astigmatic aberrations of different levels into the analyzed vortex beam simultaneously, forming a set of aberration-transformed beam patterns in different diffraction orders in one detection plane. Figure 1 shows the principle of the operation of the proposed approach. A schematic representation of the principle of operation for determining the vortex TC in a standard way using an astigmatic or tilted lens and detecting the intensity distribution in several planes (*z*_1_, *z*_2_, … and *z_j_*) is shown in the upper part of Figure 1. A thorough description of the classical methods can be found in the according works [34,35,36,37,38,39,40,41]. The proposed approach based on a multi-channel DOE matched with astigmatic aberrations of different levels (implemented using a spatial light modulator (SLM)) and the detection of intensity distribution in a single focal plane (*z*_0_) is shown in the lower part of Figure 1. A detailed description of our method (step by step along the optical beam’s propagation) is given in Section 4.

### 3.2. Simulation Results for Multi-Channel DOEs

The complex transmission function of a multi-channel DOE matched with a set of astigmatic aberrations of various types and levels had the following form:

(10) $$\tau (x,y)=\sum_{N=1}^{N_{0}}\sum_{j=1}^{K_{0}}\exp\left[ik\alpha_{j}Z_{N}\left(x,y\right)\right]\exp\left[i\left(a_{jN}x+b_{jN}y\right)\right]$$

where *N*_0_ is the number of DOE channels corresponding to astigmatic types of aberrations $Z_{n,2}(r,\varphi )$, *K*_0_ is the number of filter channels corresponding to different levels of aberrations $\alpha_{j}$, and $a_{jN},b_{jN}$ are spatial carrier parameters along the X and Y axes.

**Figure 2:**
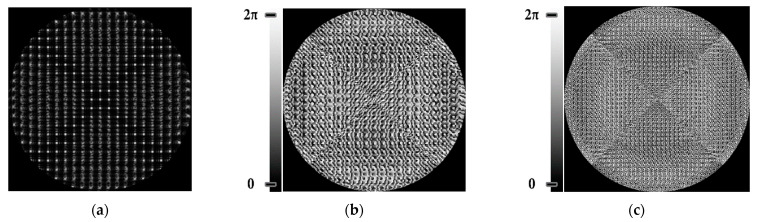

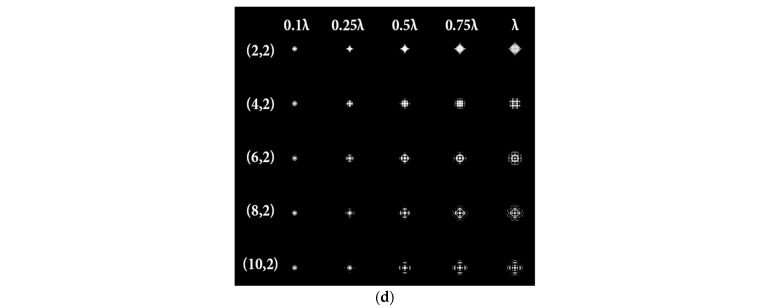
Amplitude (**a**) and phase (**b**) of a 25-channel amplitude-phase DOE, (**c**) the coded phase of DOE matched to different astigmatic aberrations $Z_{n,2}(r,\varphi )$ (*n* = 2, 4, 6, 8, 10) with various levels $\alpha_{j}$, intensity distribution in the focal plane (**d**) at the Gaussian beam illumination (correspondence of aberrated PSF to diffraction orders is shown).

Figure 2 shows the amplitude (Figure 2a) and phase (Figure 2b) of the complex transmission function for a 25-channel DOE of Equation (10) matched to *N*_0_ = 5 different astigmatic aberrations $Z_{n,2}(r,\varphi )$ (*n* = 2, 4, 6, 8, 10) with *K*_0_ = 5 different levels $\alpha_{j}$($\alpha_{1}=0.1\lambda$, $\alpha_{2}=0.25\lambda$, $\alpha_{3}=0.5\lambda$, $\alpha_{4}=0.75\lambda$, $\alpha_{5}=\lambda$).

For the optical implementation of a complex function of Equation (10) using a phase spatial light modulator (SLM), we applied the phase-coding method [66]. This method was applied to the normalized complex function |g(x,y)|≤1:

(11) $$g(x,y)=\tau (x,y){\left(\max\left|\tau (x,y)\right|\right)}^{-1}$$

where τ(x,y) was derived from Equation (10). The normalized amplitude-phase function g(x,y) was replaced with a phase function h(x,y) according to the rule:

(12) $$h(x,y)=\left\{ \begin{array}{l} \exp\left[i\arg\left[g(x,y)\right]\right],g(x,y)\geq \Delta ,\\ \exp\left[i\arg\left[g(x,y)\right]+i\mu \right],g(x,y)\leq \Delta , \end{array}\right.$$

(13) $$\mu =\left\{ \begin{array}{l} \pi ,\mathrm{sgn}\left(S_{ij}\right)>0,\\ 0,\mathrm{sgn}\left(S_{ij}\right)<0, \end{array}\right.$$

where parameter $S_{ij}\in [-0.5;0.5]$ is a pseudo-random value and *S* is the number of pseudo-random numbers from the segment [−0.5; 0.5].In a number of numerical experiments, it was found [66] that the best correspondence between the intensity distribution in the focal plane for the amplitude-phase element of Equation (10) and for the phase element of Equation (12) was achieved with the parameters of the partial coding method *S* = 5 and Δ = 0.1. Figure 2 shows the phase (Figure 2c) of the phase-coded multi-channel DOE of Equation (12).

Figure 2c shows the phase-only coded multi-channel DOE. The operating result of such a DOE in the focal plane at the Gaussian beam illumination is shown in Figure 2d; the intensity in different diffraction orders corresponded to the point spread function (PSF) for each aberration encoded in the DOE.

When the 25-channel DOE (shown in Figure 2) was illuminated with vortex beams, different astigmatic patterns formed in the focal plane. Examples of the DOE operation for vortex beams with TC *l* = 1, 3, 5 are shown in Table 5. In fact, the distributions in different diffraction orders in one line corresponded to different detection distances (*z*_1_, *z*_2_, … and *z_j_*) in the classical scheme with an astigmatic or tilted lens. Thus, to select a good level of astigmatic transformation, it was not required to move the camera. It was only necessary to select suitable diffraction orders (marked with a frame), which allowed one to clearly determine the TC from the intensity pattern.

As can be seen from the results shown in Table 5, for various values of TC, a set of “good” PSFs (marked with a box) varied. In particular, when determining the topological charge *l* = 1 (Table 5, first column), Astigmatic aberration of type (2, 2) with a level α in the range from 0.1λ to λ did not show good results, compared with other astigmatic aberrations of the type (*n*, 2). In turn, aberrations of the type (4, 2), (6, 2), (8, 2) with a level from 0.25λ to λ demonstrated the formation of sufficiently clear pictures for a visual analysis. Moreover, with an increase in the level of aberrations, the distance between the extrema became larger, which could improve the quality of determining the TC and the possibility to use machine learning methods. An aberration of type (10, 2) had a significant optical power in terms of PSF distortion and showed sufficient results only for the level α = 0.5λ.

With an increase in the TC to *l* = 3 (Table 5, second column), the use of the classical astigmatic aberration (2, 2) did not give a good result. Astigmatic distortions of the types (4, 2), (6, 2), (8, 2) and (10, 2) with level α in the range from 0.75λ to λ showed significantly better results. However, high-order aberrations, such as (8, 2) and (10, 2), with an increase in the level of α, introduced unnecessary artifacts into the PSF patterns, which could make it difficult to automatically determine the TC.

In the analysis of a vortex beam with a large topological charge *l* = 5 (Table 5, third column), good results were obtained only when using aberrations (4, 2), (6, 2) and (8, 2) with high level α = λ. Thus, with an increase in the detected TC, one could observe a tendency towards the need to increase the level α of astigmatic aberrations (*n*, 2).

For a more detailed analysis of the influence of astigmatic aberrations of type (*n*, 2) and level α, the action of multi-channel DOEs of Equation (10) was considered, consistent with one type of aberration (*N*_0_ = 1) with a large range of α from 0.1λ to 5λ. The simulation results are shown in Table 6. The diffraction orders with astigmatic PSFs, which allowed for it to be possible to clearly determine the TC, were marked with a frame. We could confirm the following trend: with an increase in the TC of the vortex beam, an increase in the level of astigmatism α was required for correct detection. In addition, it required a higher level of α to form a well-defined astigmatism pattern when using the classical type of astigmatism (2, 2) (first column of Table 6). The astigmatism of type (6, 2) (third column of Table 6) was not suitable for vortex beams with a large TC due to the appearance of redundant artifacts in the intensity pattern.

**Figure 3:**
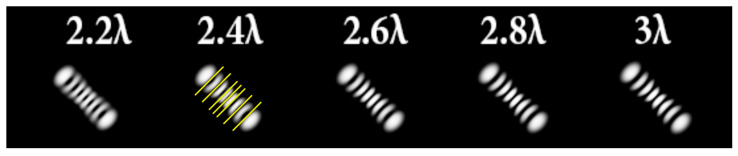
Detailed simulation results for multi-channel DOEs matched with one type of aberration $Z_{4,2}(r,\varphi )$ with α ranging from 2.2λ to 3λ when illuminated by a vortex beam with TC *l =* 7.

The best results were obtained for the astigmatic aberration of type (4, 2) (second column of Table 6). Figure 3 shows, in more detail, the simulation results for this type of aberration at a level of α from 2.2λ to 3λ, obtained by analyzing a vortex beam with TC *l* = 7. For clarity, dark intensity lines between light intensity extrema were indicated with yellow lines (the number of seven lines corresponded to *l* = 7). Figure 3 clearly shows that with an increase in the aberration level α, the distance between adjacent extrema increased, which allowed for it to be possible to more confidently detect the TC.

### 3.3. Optimization for High-Order TC

**Figure 4:**
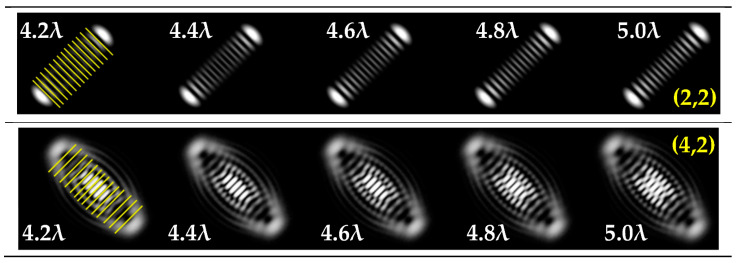
Detailed simulation results for multi-channel DOE matched with one type of aberration $Z_{2,2}(r,\varphi )$ and $Z_{4,2}(r,\varphi )$ with α ranging from 4.2λ to 5λ when illuminated with a vortex beam with TC *l =* 14.

It was noted in [46] that the use of a tilted lens for determining the TC was inefficient, especially for high orders of TC, and it was proposed to use a cylindrical lens, the efficiency of which was shown up to *l* = 100. Note that a cylindrical lens actually corresponds to the presence of two aberrations (Table 2, lines two and three): astigmatic aberration *Z*_2,±2_ and defocus *Z*_2,0_. In our approach, we proposed to replace defocusing with different levels of astigmatic aberration α. We carried out numerical studies at high TCs with various types of aberrations to determine the most appropriate transformation. The simulation results for the TC value *l* = 14 are shown in Table 7 and in Figure 4.

As can be seen from the results, the classic type of astigmatism (2, 2) (first column of Table 7) provided better pictures compared with other types of aberrations. More detailed simulation results for level α from 4.2λ to 5λ are shown in Figure 4.

Thus, as shown by the simulation results, it made sense to use high-order astigmatic aberrations *n* = 4, 6 for small values of the vortex beam’s TC. In this case, small levels of α from 1.2λ to 2λ were sufficient. However, an aberration of the type (6, 2) formed a PSF that was difficult to analyze at high values of TC. Pictures that were clearer for the analysis were provided by aberrations of the type (2, 2) and (4, 2), but at a high level (α > 4λ).

Thus, the problem in determining the TC of a vortex beam was simplified by the use of a multi-channel DOE of Equation (10), which provides a much larger amount of information in a single plane compared to the standard way based on an astigmatic or tilted lens. The proposed approach provided a more confident and correct detection of TCs without the need to make adjustments to the optical system or register the intensity in several planes.

## 4. Laboratory Experiments

**Figure 5:**
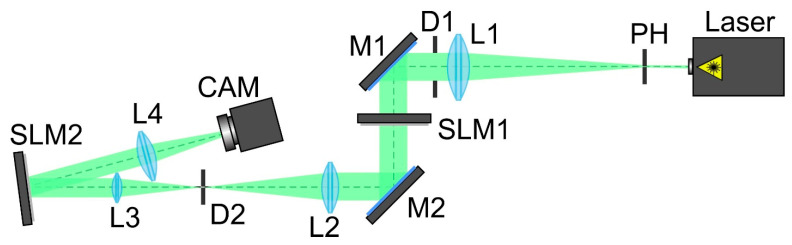
The experimental setup for detecting the TC of a vortex beam using a multi-channel DOE. Laser is a solid-state laser (λ = 532 nm); PH is a pinhole (hole size of 40 μm); L1, L2, L3 and L4 are spherical lenses (*f*_1_ = 350 mm, *f*_2_ = 300 mm, *f*_3_ = 200 mm and *f*_4_ = 250 mm); SLM1 is a transparent spatial light modulator (HOLOEYE LC 2012); SLM2 is a reflective spatial light modulator (HOLOEYE PLUTO VIS); D1 and D2 are circular apertures; M1 and M2 are mirrors; CAM is a ToupCam UCMOS08000KPB video camera.

To confirm the effectiveness of the proposed approach and the numerical results for multi-channel DOEs matched with various types of astigmatic aberrations, we carried out experiments using one (transparent) SLM to generate vortex beams and another (reflective) SLM to implement multi-channel DOEs. The optical scheme used in the experiment is shown in Figure 5.

The scheme of the experimental setup for detecting the TC of a vortex beam using a multi-channel DOE of Equation (10) consisted of the following elements: the laser was a solid-state laser (λ = 532 nm); the PH was a pinhole (hole size 40 microns); L1, L2, L3 and L4 were spherical lenses (*f*_1_ = 350 mm, *f*_2_ = 300 mm, *f*_3_ = 200 mm and *f*_4_ = 250 mm); SLM1 was a transparent spatial light modulator (HOLOEYE LC 2012) for a vortex beam generation; SLM2 was a reflective spatial light modulator (HOLOEYE PLUTO VIS) for a multi-channel DOE implementation; D1 and D2 were circular apertures; M1 and M2 were mirrors; CAM was a video camera (ToupCam UCMOS08000KPB).

The laser radiation of a solid-state laser (λ = 532 nm) was collimated using a system consisting of a pinhole (PH) with an aperture diameter of 40 μm and a spherical lens (L1). A circular aperture D1 was used to separate the central light spot from the surrounding light and dark rings that occurred during the pinhole diffraction.

Then, the laser beam expanded and reflected from mirror M1 passed through a transparent SLM1 (HOLOEYE LC 2012 with a resolution of 1024 × 768 pixels and a pixel size of 36 μm), which was used to generate a vortex beam. Lenses L2 and L3 and aperture D2 were used to spatially separate the laser beam formed with the first modulator and the unmodulated transmitted zero-order laser beam.

Mirror M2 was used to direct the shaped laser beam to the display of the second modulator. A reflective SLM2 (HOLOEYE PLUTO VIS with a resolution of 1920 × 1080 pixels and a pixel size of 8 µm) was used to implement the phase-coded multi-channel DOE of Equation (12). The used encoding procedure was described in detail in Section 3.2. It served to determine the TC of the vortex beam. The laser beam reflected from SLM2 was directed into the lens L4 (*f*_4_ = 250 mm), which focused it onto the video camera (ToupCam UCMOS08000KPB CAM camera with a resolution of 3264 × 2448 and a pixel size of 1.67 µm).

**Figure 6:**
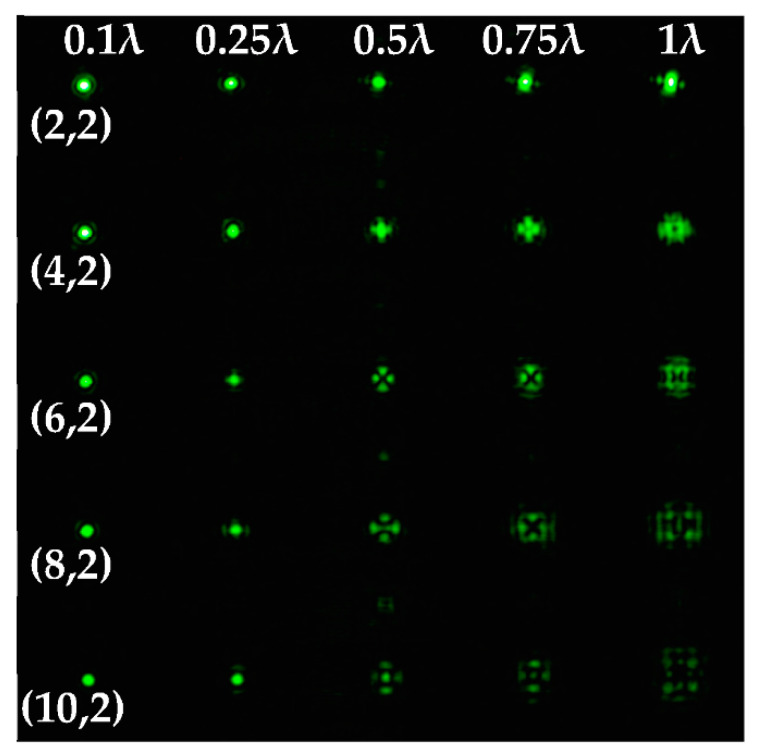
The experimentally registered intensity distribution for the Gaussian beam illumination of the multi-channel DOE matched with different astigmatic aberrations of type (*n*, 2) of different levels of α from 0.1λ to λ.

The experimentally registered intensity distribution for the Gaussian beam illumination of the multi-channel DOE matched with different astigmatic aberrations of type (*n*, 2) of different levels of α from 0.1λ to λ is shown in Figure 6. The results corresponded to the numerical results shown in Figure 2d. The action of this DOE under vortex beam illumination with different TC values (*l* = 1, 2, 3, 5) is shown in Table 8. Diffractive orders with astigmatic intensity pictures convenient for TC recognition were marked with frames.

As a result of the conducted optical experiment, it was found that the classical astigmatic aberration of the type (2, 2) with a level in the range from 0.1λ to λ allowed forit to be possible to detect only small TC values (|*l*| = 1) of the vortex beam. In addition, astigmatic aberrations of type (*n*, 2) for 2 ≤ *n* ≤ 10 allowed for it to be possible to visualize TC *l* = 1 with a weak aberration level (α = 0.1λ). There was a trend towards the successful use of higher-order astigmatic aberrations (*n* = 4, 6, 8) with an increase in the analyzed TC orders. The best results were shown with the (4, 2) astigmatic aberration for all considered TC orders, as it was predicted in the numerical results.

## 5. Discussion

By analyzing the numerical and experimental results, we could conclude that the astigmatic aberration of the type (4, 2) with a level of λ (and higher) provided the successful TC detection of a vortex beam at least up to *l* = 5 (confirmed with the experimental results) and potentially up to *l* = 14 (according to the numerical results). Therefore, it was a good alternative to the classical astigmatism (2, 2).

The proposed approach greatly facilitated the analysis of the characteristics of a vortex beam based on measurements in a single plane without additional adjustments. Thus, the proposed multi-channel DOEs provided a simple method for a vortex TC analysis using variable astigmatic transformations. Confident detection could be achieved using wave aberrations of the *Z_n,±_*_2_ type and their combination.

It can be seen from the intensity distribution in the focal plane after the astigmatic transformation of the vortex Laguerre–Gaussian modes with a TC value *l* into the Hermite–Gaussian modes with orders (*p*, *l*) = (0, *l*) [67,68,69] that the TC of the vortex beam was visualized in the intensity of the astigmatically transformed beam.

It is worth noting that there was a difference between the experimental and numerical results. One of the main reasons for this was related to the SLM shortcomings. It was possible to design a single DOE matched with a rather large number of transmission functions. However, in this case, we had to conduct amplitude encoding to obtain a pure phase element [70]. When a SLM was used for a multichannel DOE implementation, there were a number of limitations, primarily due to the resolution (in particular, HOLOEYE PLUTO VIS had a 1920 × 1080 pixel resolution with an 8 µm pixel size). However, a phase multichannel DOE implemented with the etching method could be fabricated with a 1 µm pixel size. The successful application of these 32- and 64-channel DOEs was shown in the analysis of the LP modes of a step-index fiber [71].

The discrepancy seen between the experimental and modeling results could also be explained through the distortions of the initial vortex beams caused by astigmatism resulting from imperfections in the optical elements. It is well known that even weak astigmatism can distort the annular-shaped vortex beam [46]. However, even these distortions allow one to use the designed multi-channel DOEs for the determination of present aberrations.

The authors planned their further research in terms of determining the topological charge with super-imposed optical vortices or the super-position of several vortices. In this case, the astigmatism pattern would be more complex, so further processing, including data mining, would likely be required for the analysis.

## 6. Conclusions

A detailed numerical and experimental study for the possibility of detecting and analyzing the topological charge of a vortex beam by introducing aberrations of various types and levels into analyzed vortex beams was realized. The numerical results of the successful detection of the topological charge of a vortex beam (up to *l* = 14) using multi-channel DOEs matched with astigmatic aberrations were shown.

The possibility of determining the topological charge by introducing wave aberrations of the astigmatic type with different weight coefficients was shown. Moreover, reliable detection could be achieved by using wave aberrations of the *Z_n,±_*_2_ type and their combination with defocusing aberrations, which was confirmed with the analytical representation of these aberrations as a super-position of Zernike functions.

The proposed method based on multi-channel DOEs matched with different types and levels of astigmatic aberrations provided different beam transformations simultaneously and formed a set of aberration-transformed patterns in different diffraction orders in one detection plane. This simplified the experimental detection of vortex TCs in comparing to the classical method with a tilted lens, because there was no need for optical system tuning (change in the tilt angle or distance of the detecting plane).

Moreover, this method allows for one to obtain much more information at a single detection, which can be useful in the analysis of intensity patterns by means of data mining and convolutional neural networks [72,73,74,75,76].

## Figures and Tables

**Table 1:** Zernike functions of Equation (5) corresponding to astigmatic aberrations.

*n*	*m*	Aberration Type	Mathematical Representation	Phase
2	2	Astigmatism	$\sqrt{6}r^{2}\cos(2\varphi )=c_{1,1}(x^{2}-y^{2})$	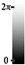 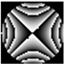
4	2	Fourth order astigmatism	$\sqrt{10}(4r^{4}-3r^{2})\cos(2\varphi )=c_{2,1}(x^{4}-y^{4})-c_{2,2}(x^{2}-y^{2})$	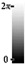 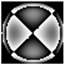
6	2	Sixth order astigmatism	$\begin{array}{l} \sqrt{14}(15r^{6}-20r^{4}+6r^{2})\cos(2\varphi )=\\ c_{3,1}(x^{4}-y^{4})(x^{2}+y^{2})-c_{3,2}(x^{4}-y^{4})+c_{3,3}(x^{2}-y^{2}) \end{array}$	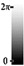 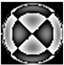
8	2	Eighth order astigmatism	$\begin{array}{l} \sqrt{18}(56r^{8}-105r^{6}+60r^{4}-10r^{2})\cos(2\varphi )=\\ c_{4,1}x^{8}+c_{4,2}x^{6}y^{2}-c_{4,3}x^{6}-c_{4,4}x^{4}y^{2}+c_{4,5}x^{4}-c_{4,6}x^{2}y^{6}+\\ +c_{4,7}x^{2}y^{4}-c_{4,8}x^{2}-c_{4,9}y^{8}+c_{4,10}y^{6}-c_{4,11}y^{4}+c_{4,12}y^{2} \end{array}$	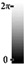 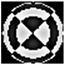
*q*	2	*q*th order astigmatism	$\sqrt{\frac{q+1}{\pi }}\sum_{s=0}^{q/2-1}\frac{{\left(-1\right)}^{p}\left(q-s\right)!}{s!\left(q/2+1-s\right)!\left(q/2-1-s\right)!}{\left(\frac{r}{r_{0}}\right)}^{q-2s}\cos\left(2\varphi \right)$	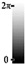 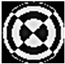

**Table 2:** Correspondence of some types of astigmatic transformations of Equation (4) and Zernike functions.

Astigmatic Transformation Equation (4)	Mathematical Representation as Zernike Functions of Equation (5)	Phase
*xy*	$r\cos(\varphi )\cdot r\sin(\varphi )=d_{1,1}Z_{2,-2}$	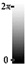 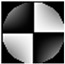
$x^{2}$	$r^{2}\cos^{2}(\varphi )=d_{2,1}Z_{2,0}+d_{2,2}Z_{2,2}$	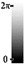 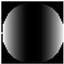
$y^{2}$	$r^{2}\sin^{2}(\varphi )=d_{3,1}Z_{2,0}-d_{3,2}Z_{2,2}$	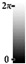 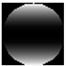
$x^{2}-y^{2}$	$r^{2}\cos^{2}(\varphi )-r^{2}\sin^{2}(\varphi )=d_{4,1}Z_{2,2}$	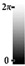 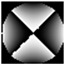
${\left(x-y\right)}^{2}$	$r^{2}\cos^{2}(\varphi )-2r^{2}\cos(\varphi )\sin(\varphi )+r^{2}\sin^{2}(\varphi )=d_{5,1}Z_{2,0}-d_{5,2}Z_{2,-2}$	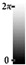 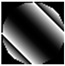
${\left(x+y\right)}^{2}$	$r^{2}\cos^{2}(\varphi )+2r^{2}\cos(\varphi )\sin(\varphi )+r^{2}\sin^{2}(\varphi )=d_{6,1}Z_{2,0}+d_{6,2}Z_{2,-2}$	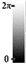 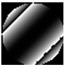

**Table 3:** Simulation results of astigmatic transformations of vortex beams $\Psi_{0,l}(r,\varphi )$ with aberrations of the form $\exp\left[ik\alpha Z_{n,2}(r,\varphi )\right]$ in the focal plane.

Astigmatic Parameters	Topological Charge					
	*l* = −5	*l* = −3	*l* = −1	*l* = 1	*l* = 3	*l* = 5
*n* = 2, α = 3λ	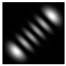	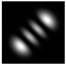	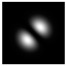	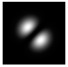	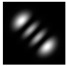	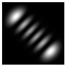
*n* = 4, α = λ	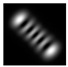	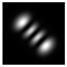	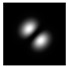	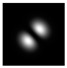	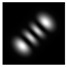	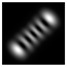
*n* = 6, α = λ	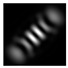	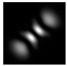	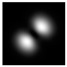	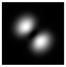	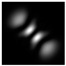	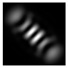
*n* = 8, α = λ	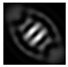	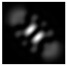	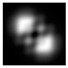	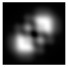	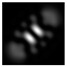	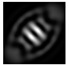

**Table 4:** Simulation results of astigmatic transformation of vortex beams $\Psi_{0,l}(r,\varphi )$ with aberrations of the form $\exp\left[ik\alpha Z_{n,2}(r,\varphi )\right]$ at various distances Δ*z* from the focal plane.

$\mathrm{Vortex}\;\mathrm{Beam}\;\Psi_{0,l}(r,\varphi )$	Astigmatic Parameters	Intensity Distributions at Various Distances Δ*z* from the Focal Plane			
		0 mm	100 mm	200 mm	300 mm
TC *l* = 1 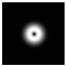 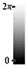 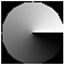	*n* = 2, α = 3λ	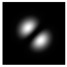	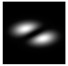	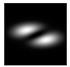	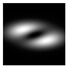
	*n* = 4, α = λ	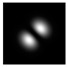	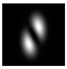	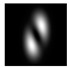	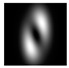
	*n* = 6, α = λ	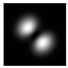	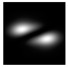	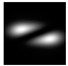	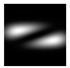
TC *l* = 3 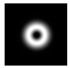 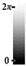 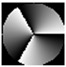	*n* = 2, α = 3λ	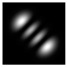	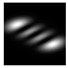	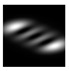	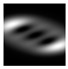
	*n* = 4, α = λ	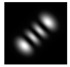	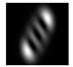	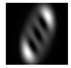	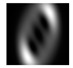
	*n* = 6, α = λ	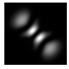	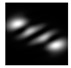	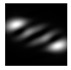	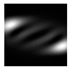
TC *l* = 5 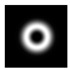 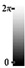 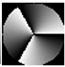	*n* = 2, α = 3λ	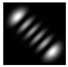	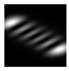	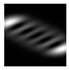	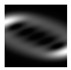
	*n* = 4, α = λ	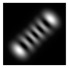	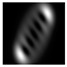	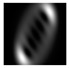	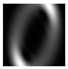
	*n* = 6, α = λ	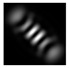	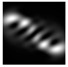	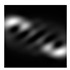	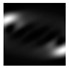

**Table 5:** The action of the 25-channel DOE illuminated with vortex beams with a TC *l* = 1, 3, 5.

*l* = 1	*l* = 3	*l* = 5
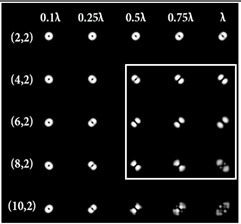	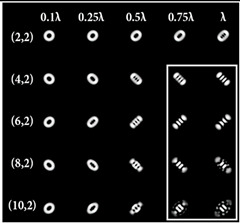	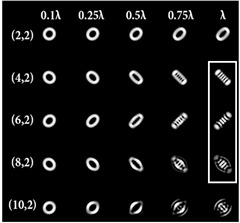

**Table 6:** Simulation results for multi-channel DOEs matched with one type of aberration $Z_{n,2}(r,\varphi )$ (*n* = 2, 4, 6) with α ranging from 0.2λ to 5λ when illuminated with a vortex beam with a TC *l* = 3, 5, 7.

*Vortex TC* ***l*** **= 3**		
*n* = 2	*n* = 4	*n* = 6
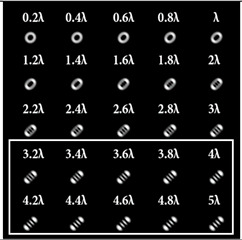	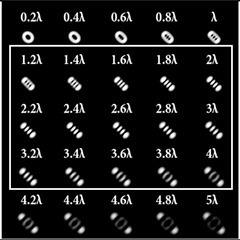	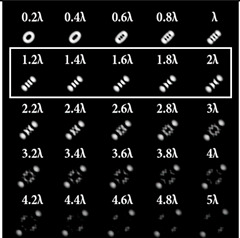
*Vortex TC* ***l* = 5**		
*n* = 2	*n* = 4	*n* = 6
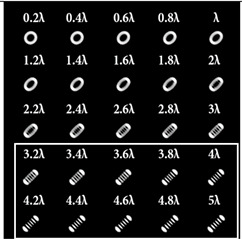	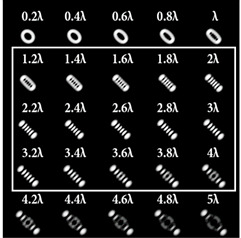	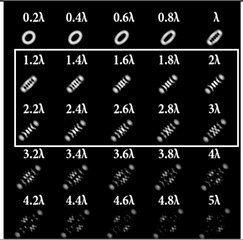
*Vortex TC* ***l* = 7**		
*n* = 2	*n* = 4	*n* = 6
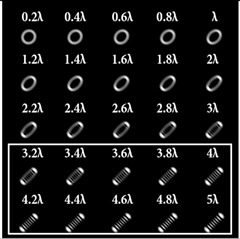	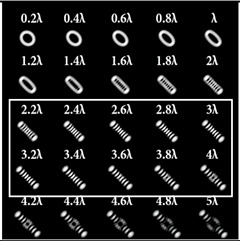	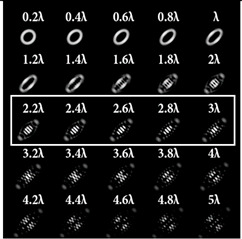

**Table 7:** Simulation results for multi-channel DOE matched with one type of aberration $Z_{n,2}(r,\varphi )$ (*n* = 2, 4, 6) with α ranging from 0.2λ to 5λ when illuminated with a vortex beam with TC *l* = 14.

*n* = 2	*n* = 4	*n* = 6
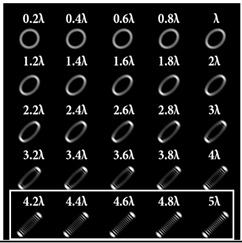	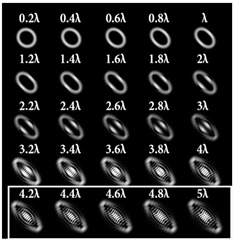	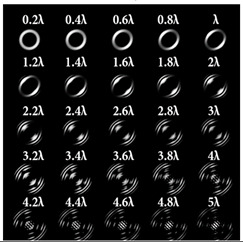

**Table 8:** The experimental results for the 25-channel DOE when illuminated with a vortex beam with TC *l* = 1, 2, 3, 5 (diffractive orders with astigmatic intensity pictures convenient for TC recognition were marked with frames).

*TC*	25-Channel DOE Action	*TC*	25-Channel DOE Action
*l* = 1	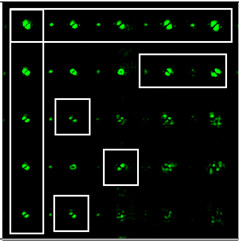	*l* = 2	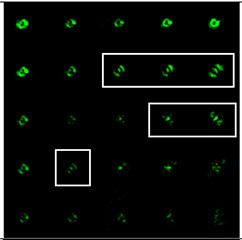
*l* = 3	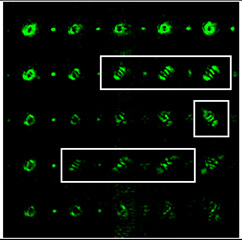	*l* = 5	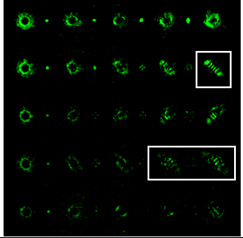

## Data Availability

Not applicable.
